# Effect of Cataracts on Hydroxychloroquine Retinopathy Screening

**DOI:** 10.3390/diagnostics15212736

**Published:** 2025-10-28

**Authors:** Ji Soo Kang, Seong Joon Ahn, Yu Jeong Kim

**Affiliations:** Department of Ophthalmology, Hanyang University Hospital, Hanyang University College of Medicine, Seoul 04763, Republic of Korea

**Keywords:** cataracts, hydroxychloroquine retinopathy, optical coherence tomography, fundus autofluorescence, Humphrey visual fields

## Abstract

**Background/Objectives:** To evaluate the modality-specific impact of cataracts on the detection of hydroxychloroquine retinopathy. **Methods:** In this retrospective cohort study, 202 eyes (101 patients) with confirmed HCQ retinopathy were included; analyses focused on 141 cataractous eyes from 72 patients. At each visit, the severity of cataracts in 141 eyes was graded using the Lens Opacities Classification System III (LOCS III), with clinically significant cataracts defined as a LOCS III grade ≥ 3. Screening was performed using swept source optical coherence tomography (OCT), ultrawide field fundus autofluorescence (FAF), and Humphrey visual field (HVF) tests. The detection rates of abnormalities on OCT, FAF, and HVF were compared between minimal (at the time of diagnosis or after cataract surgery) and maximal cataract severity as well as between eyes with clinically significant cataracts and others. Multivariate logistic regression was performed to identify the factors associated with the detection of retinopathy-associated abnormalities across each screening modality. **Results:** Of the 141 eyes with cataracts, 52 (36.9%) developed clinically significant opacities during the monitoring period, and 23 (16.3%) underwent cataract surgery. OCT detected ellipsoid zone disruptions in 100% of cataractous eyes, while visual fields revealed characteristic paracentral scotomas with comparable sensitivity regardless of cataract severity. In contrast, FAF sensitivity was significantly lower in eyes with clinically significant cataracts (61.5%) compared to those with mild cataracts (92.1%, *p* < 0.001). Sensitivities were also reduced at maximal versus minimal severity in eyes with clinically significant cortical opacities and nuclear opalescence (both *p* < 0.05). Multivariate analysis demonstrated that higher cortical opacity (odds ratio [OR] 0.43 per grade increase, 95% CI 0.22–0.85) and nuclear opalescence (OR 0.21, 95% CI 0.07–0.66) independently decreased FAF detection, whereas greater retinopathy severity was positively associated with detection on both FAF (OR 4.85, 95% CI 1.40–16.9) and HVF (OR 3.37, 95% CI 1.17–9.71). **Conclusions:** Cataracts impaired the FAF-based detection of hydroxychloroquine retinopathy, while OCT and HVF remained reliable despite significant lens opacities. Therefore, clinicians should consider cataract severity when interpreting FAF results and prioritize OCT and HVF assessments in patients with clinically significant cataracts.

## 1. Introduction

Hydroxychloroquine (HCQ)-induced retinal toxicity, which primarily affects the photoreceptors in the parafoveal and pericentral regions of the eye, is a potentially vision-threatening adverse effect from the treatment of chronic autoimmune conditions such as systemic lupus erythematosus (SLE) and rheumatoid arthritis (RA) that also presents a diagnostic challenge as the treatment durations for these conditions continue to increase [1]. Although modern dosing guidelines (≤5 mg/kg real body weight daily) have reduced the risk of retinopathy, the cumulative risk escalates dramatically beyond 10 years of use—affecting 20–50% of patients by 20 years—depending on the daily dose-to-body weight ratio [1,2,3]. A recent study found a positive correlation between central corneal thickness and prolonged HCQ use, underscoring the need for comprehensive ocular assessment in HCQ-treated patients [4].

For the retinal toxicity, the American Academy of Ophthalmology (AAO) 2016 guidelines recommend baseline evaluations within 1 year of the initiation of HCQ, and annual screenings after 5 years of treatment [1]. Multimodal assessments—combinations of spectral-domain optical coherence tomography (SD-OCT), fundus autofluorescence (FAF), and automated perimetry (10-2 or wider Humphrey visual field [HVF] test)—are the diagnostic cornerstone of HCQ screening, with SD-OCT detecting parafoveal or pericentral photoreceptor and retinal pigment epithelium defects, HVF showing paracentral ring scotomas corresponding to outer retinal defects, and FAF revealing hyper- or hypoautofluorescent rings [5,6].

Patients being treated with HCQ are often also taking systemic corticosteroids, a well-known risk factor for the formation of cataracts [7], which may be a significant issue among these patients and potentially affect the accuracy of screening test results. Cataracts result in wavelength-dependent light scattering that can impact ophthalmic imaging such as OCT and FAF [8,9]. This is particularly concerning, as these modern imaging modalities are critical when screening for HCQ retinopathy, providing objective assessments of HCQ-induced retinal damage and allowing for the early detection of HCQ retinopathy. We hypothesized that cataracts may impact the performance of each screening modality and that the degree of lens opacity may correlate with detection sensitivity across modalities and disease stages.

This study aimed to evaluate the effects of cataracts on HCQ retinopathy screening modalities through a detailed analysis of 74 patients diagnosed with both HCQ retinopathy and cataract. Through correlating detection across each modality with lens opacity and retinopathy stage, we assessed cataracts’ impact on multimodal screening and formulated recommendations for interpreting results in cataractous patients.

## 2. Materials and Methods

### 2.1. Patients

This retrospective cohort study analyzed 101 patients diagnosed with HCQ retinopathy between 1 January 2016 and 31 March 2025 at a tertiary referral center. The inclusion criteria were as follows: patients with HCQ retinopathy and complete multimodal datasets (OCT, FAF, and HVF). The exclusion criteria included the absence of a cataract evaluation (no grade documentation of cataract status), media opacity other than cataract (i.e., corneal or vitreous opacity), or history of cataract extraction before the initial screenings. Supplemental Figure S1 shows the inclusion and exclusion criteria for this study and the number of patients or eyes excluded per criterion. Demographic data including age, sex, and primary diagnosis for HCQ use were recorded. This study was performed in accordance with the tenets of the Declaration of Helsinki and was approved by the Institutional Review Board of Hanyang University Hospital (Approval No. 2025-09-010). The requirement for informed consent was waived because the study used de-identified, retrospective clinical data.

### 2.2. Screening and Monitoring Examinations

Swept-source OCT imaging was acquired using a DRI-Triton (Topcon Inc., Tokyo, Japan) with a 12 × 9 mm^2^ volume scan. The OCT images were analyzed for ellipsoid zone defects, distinguishing between parafoveal (500–1500 μm or approximately 2–6° from the fovea, respectively) and pericentral (>1500 μm from the fovea) abnormalities [10]. To ensure sufficient signal strength and clarity for reliable analysis, only OCT images with an image-quality score greater than 60 were included in the analyses. Ultrawide-field fundus FAF imaging was performed using an Optos 200Tx confocal scanning laser ophthalmoscope (Optos PLC, Dunfermline, UK) with a 200° field of view. FAF excitation was provided by a 532 nm laser, and autofluorescence emission was collected with a barrier filter above 540 nm. Images were captured in single-shot high-resolution mode using the manufacturer’s default laser power and detector-gain settings; only those with optimal focus, minimal motion artifact, and the highest contrast were selected for analysis. Standard automated perimetry was conducted using the Swedish Interactive Threshold Algorithm 30-2 and/or 10-2 strategies on a Humphrey Field Analyzer II or III (Carl Zeiss Meditec Inc., Dublin, CA, USA). Only reliable visual field tests (fixation loss < 20%, false-positive rate < 15%, false-negative rate < 15%) were included in our analyses, and the FAF images with the highest contrast and clarity were selected from each visit. Both reviewers (SJA and JSK) independently reviewed all images and test results, and any discrepancies were resolved by consensus.

### 2.3. Diagnostic and Classification Criteria

HCQ retinopathy was diagnosed as defined in previous studies and the AAO guidelines [1,11], requiring at least two structural and functional findings including: (1) OCT demonstrating a parafoveal or pericentral photoreceptor (ellipsoid zone) disruption, (2) FAF revealing a hyper- or hypo-autofluorescent circumferential (ring-like) lesion, and/or (3) functional evidence defined as a patchy or paracentral ring scotoma, central island, or a whole defect on the HVF pattern deviation map [12] corresponding to the structural defects. Retinopathy patterns were classified as parafoveal, pericentral, or mixed (both parafoveal and pericentral), and severity staging (early, moderate, and severe) was based on modified AAO guidelines using OCT and FAF features [6].

Cataracts were classified using the Lens Opacities Classification System III (LOCS III) [13], and more specifically, based on nuclear opalescence (NO), cortical opacity (C), and posterior subcapsular cataracts (P). Cataract grading was performed under pharmacologic mydriasis by a single experienced grader (S.J.A.) using the predefined LOCS III criteria described above, and cataracts with any LOCS III grade ≥ 3 were considered clinically significant [14]. Images acquired at the maximum cataract grade—defined as the highest grade recorded at the most recent visit or immediately before cataract surgery—were compared with those obtained at the minimum cataract grade, either at the time of HCQ retinopathy diagnosis in eyes without cataract surgery or postoperatively in eyes that underwent extraction following HCQ retinopathy diagnosis.

### 2.4. Statistical Analyses

Patient demographic data, HCQ usage details, and clinical retinopathy characteristics were analyzed using descriptive statistics. Categorical variables were compared using Fisher’s exact or the Chi-squared test as appropriate, while paired comparisons of detection rates across different visits were conducted using McNemar’s test. Normality of continuous variables was evaluated using the Shapiro–Wilk test. According to the results, comparisons of clinical parameters were performed using either Student’s *t*-test or the Mann–Whitney test. Multivariate logistic regression was performed to identify which clinical factors—age, sex, HCQ indication, retinopathy severity and pattern, and cataract grade—were associated with the detection of retinopathy by each screening modality. Statistical analyses were performed using SPSS version 27.0 (IBM Corp., Armonk, NY, USA), with statistical significance set at *p* < 0.05.

## 3. Results

### 3.1. Demographic and Clinical Characteristics

Among the 101 patients in the HCQ retinopathy cohort, 80 (79.2%) had a cataract in at least one eye or had undergone cataract extraction prior to the time of retinopathy diagnosis, yielding an overall prevalence of 79.2%. Of the 202 eyes, 16 (7.9%) had undergone cataract extraction before being diagnosed with retinopathy, cataract status was unknown for 2 (1.0%), and 43 (21.3%) had no diagnosed cataracts. Among the 200 eyes with a documented lens status during the post-retinopathy monitoring period, 141 eyes (70.5%), which were included in subsequent analyses, had cataracts.

Table 1 presents the demographic and clinical characteristics of the included patients with HCQ retinopathy, showing that an overwhelming majority of the patients (94.4%) were female, with an average age of 60.8 ± 12.4 years. Among these patients, the most common indications for HCQ therapy were RA (37 patients) and SLE (31 patients). The mean daily dose of HCQ was 261.8 mg, which corresponded to an average of 5.0 mg/kg of real body weight, while 41.7% of the patients were on a regimen that exceeded 5 mg/kg. The duration of HCQ use averaged 190.6 months (range, 24–372 months). The cumulative dose reached an average of 1487 g, with a high proportion (80.6%) of these patients having had a past medical history of systemic corticosteroids.

### 3.2. Presence or Severity of Cataracts and Screening Test Results

Table 2 demonstrates the clinical details of the cataracts of the included patients. Clinically significant cataracts were noted in 52 of 141 (36.9%) eyes. Nuclear cataracts ≥ grade 2 were observed in 84 (59.6%) eyes, whereas cortical and posterior capsular cataracts (≥grade 1) were observed in 62.4% and 20.6% of the eyes, respectively. Table 2 also shows the maximum LOCS III grades for nuclear opalescence, cortical opacity, and posterior capsular opacity at the most recent visit or at the last visit prior to cataract removal, which was performed in 23 eyes (16.3%) during the observation period.

**Figure 1:**
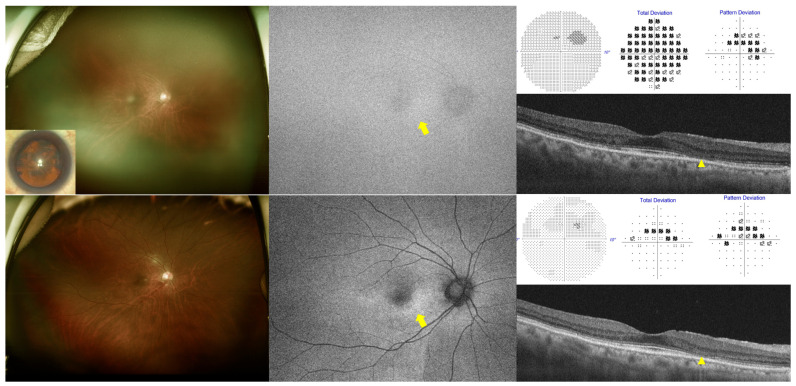
Multimodal imaging findings in a 67-year-old female with hydroxychloroquine retinopathy and cataract (LOCS III: NO3C4P0), shown before (**top**) and after (**bottom**) cataract surgery. **Left**: ultra-widefield fundus photograph (inset: anterior segment image showing pre-surgical cataract); **middle**: fundus autofluorescence (FAF); **right upper**: grayscale, total deviation, and pattern deviation maps from Humphrey 10-2 visual field tests; **right lower**: optical coherence tomography (OCT). Yellow arrows indicate areas of FAF that were obscured before cataract surgery and revealed parafoveal hyperautofluorescence after surgery. Arrowheads highlight ellipsoid zone loss on the corresponding OCT images.

Figure 1 and Supplemental Figure S2 provide photographic examples illustrating the effects of cataracts on each screening modality (OCT, FAF, and HVF) for detecting HCQ retinopathy. OCT consistently demonstrated outer retinal abnormalities both before and after cataract surgery. HVF also yielded consistent functional findings. Despite cataract-induced depression of total deviation maps, pattern deviation maps reliably demonstrated characteristic paracentral full- or partial-ring scotomas, even in cases of advanced cataracts. However, Figure 1 shows no definite FAF abnormalities before cataract surgery, while the FAF image in Supplemental Figure S2 is obscured by a severe posterior subcapsular cataract shadow, preventing identification of any abnormalities.

**Figure 2:**
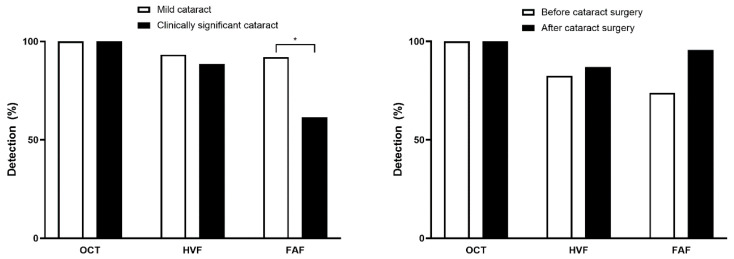
Detection rates of hydroxychloroquine retinopathy by optical coherence tomography (OCT), Humphrey visual field (HVF), and fundus autofluorescence (FAF) in eyes with mild versus clinically significant cataract (**left**) and pre- versus post-cataract surgery (**right**). Asterisk indicates statistical significance (*p* < 0.05).

Figure 2 illustrates the detection performance of each modality in patients with mild vs. clinically significant cataracts. In cases with clinically significant cataracts, the detection rate of HCQ retinopathy using FAF was significantly different, with 92.1% for mild cataracts and 61.5% for clinically significant cataracts (*p* < 0.001 by Chi-squared test). Of note, 23 eyes underwent cataract surgery, after which the proportion of eyes with detectable abnormalities on FAF increased from 73.9% to 95.7% (Figure 2, right panel), although this difference was not statistically significant (*p* = 0.074 by McNemar’s test). In contrast, detection rates for OCT remained unchanged (100% before and after surgery) while HVF showed only a minimal increase (from 82.6% preoperatively to 87.0% postoperatively). The mean OCT image-quality index was significantly lower in eyes with clinically significant cataract than in those without, but all included scans exceeded the >60 inclusion threshold (Supplemental Figure S3), indicating sufficient quality for reliable structural analysis.

### 3.3. Cataract Type and Screening Test Results

**Figure 3:**
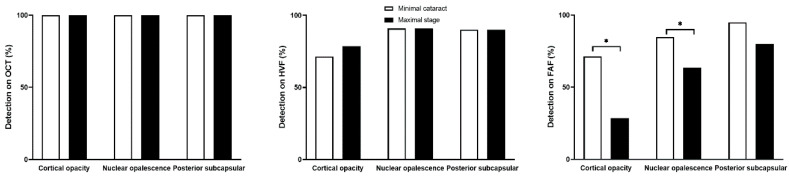
Sensitivity of optical coherence tomography (OCT), Humphrey visual field (HVF), and fundus autofluorescence (FAF) at the maximal stage of clinically significant cataracts across cortical, nuclear, and posterior subcapsular types, demonstrating a more pronounced decline in FAF sensitivity in eyes with cortical and nuclear cataract. Asterisk indicates statistical significance (*p* < 0.05).

Supplemental Figure S4 presents the prevalence of clinically significant cataracts by type and stratified by age. There were no significant differences between patients under 65 and those 65 or older in the prevalence of posterior subcapsular cataract (*p* = 0.757) or cortical opacities (*p* = 0.221), whereas nuclear opalescence was significantly more common in the older patients (*p* = 0.001). Figure 3 illustrates the effects of each type of clinically significant cataract on FAF sensitivity, revealing a more marked decrease at the maximum stage (28.6%) as compared to the minimum stage (71.4%) in eyes with clinically significant cortical opacities (*p* = 0.041). Eyes with clinically significant nuclear opalescence showed a moderate reduction in FAF detection at the maximum stage of cataracts (84.8% to 63.6%; *p* = 0.023), whereas clinically significant posterior subcapsular cataract did not impact detection (95% vs. 80%; *p* = 0.248). OCT and HVF detection rates were unaffected by cataract severity (all *p* > 0.05).

### 3.4. Association Between Screening Test Results and HCQ Retinopathy Severity in Cataract Patients

**Figure 4:**
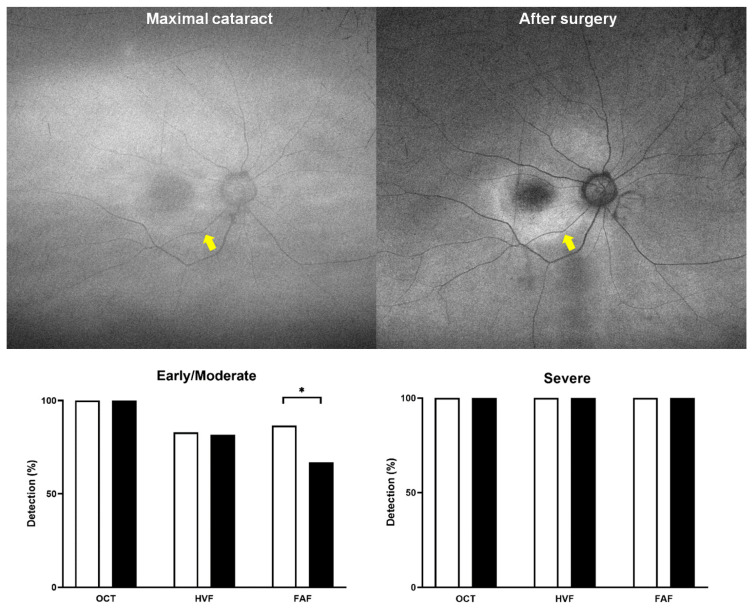
Photographic examples showing the different effects of cataract for detection of hyper- and hypo-autofluorescence on fundus autofluorescence imaging (**top**) and bar graphs comparing the sensitivities of OCT, FAF, and HVF in eyes with early/moderate (**bottom left**) and severe retinopathy (**bottom right**) between minimal (white bars) and maximal (black bars) cataract severity. Both indicate that severe stage (hypoautofluorescence) is unaffected by cataract or modality but early or moderate stage, as hyper-autofluorescence on FAF (yellow arrows), is significantly affected by maximal cataract severity (*p* < 0.001). Asterisk indicates statistical significance (*p* < 0.05).

Figure 4 demonstrates the impact of cataracts on FAF findings, which varied depending on the severity of the lesion. For example, hyperautofluorescence was greatly attenuated and barely identifiable in eyes with severe cataracts, as shown by the pre- and post-cataract removal comparison, whereas hypoautofluorescence remained identifiable before and after cataract removal. These findings demonstrate that significant cataracts impede early detection of HCQ toxicity by FAF, as lens opacities selectively attenuate hyperautofluorescent signals. In the bar graph (Figure 4, bottom), eyes with severe retinopathy did not show a decrease in the time of maximum cataract, whereas those with early or moderate retinopathy showed a significant decrease in detection sensitivity when using FAF (*p* < 0.001).

### 3.5. Factors Associated with Toxicity Detection Among Patients with Cataracts

Multivariate logistic regression (Table 3) was performed to assess FAF and HVF-based retinopathy detection. The analysis revealed that each one-grade increase in cortical opacity was significantly associated with a reduction in the odds ratio (OR) for detecting FAF abnormalities (0.43 per grade increase, 95% confidence interval [CI] = 0.22–0.85; *p* = 0.015). Additionally, each one-grade increase in nuclear opalescence was associated with a decreased OR for FAF detection (0.21 per grade increase, 95% CI = 0.07–0.66; *p* = 0.007). In contrast, neither cortical opacity nor nuclear opalescence significantly affected HVF detection rates (both *p* > 0.05). However, the severity of retinopathy significantly influenced both FAF detection (OR 4.85, 95% CI 1.40–16.9) and HVF detection (OR 3.37, 95% CI 1.17–9.71).

## 4. Discussion

This study comprehensively evaluated how cataracts affect multiple screening modalities for HCQ retinopathy and found that each imaging technique showed different sensitivities in eyes with lens opacities. OCT maintained high diagnostic reliability despite significant cataracts, whereas FAF detection declined as cataract severity increased. Thus, cataract type and severity exerted modality-specific effects on detecting retinal toxicity.

This study included patients with confirmed HCQ retinopathy, reflecting the typical demographic profile of long-term HCQ users (predominantly female, older age, and frequent corticosteroid exposure). Given this design, the findings primarily inform diagnostic interpretation in eyes with established retinopathy, rather than general screening sensitivity among all HCQ users. However, population studies have demonstrated that cataract prevalence increases sharply after age 60, with >50% of individuals in this age group exhibiting clinically significant lens opacities [15]. Our cohort had a mean age of 60.8 years, and more than 80% of patients had also been treated with systemic corticosteroids, a reflection of the standard of care in rheumatologic diseases such as SLE, for which low-dose glucocorticoids remain a mainstay of treatment. As such, the age profile and concomitant use of corticosteroids of our cohort underscored the high baseline risk for cataracts among our patients.

Accordingly, cataracts, which may significantly affect their vision and potentially require retinopathy screening, were common among our cohort of patients with HCQ retinopathy. In total, 79.2% of the patients had cataracts or prior cataract removal surgery, with 36.9% of the included eyes meeting the diagnostic criteria for clinically significant cataracts. Nuclear sclerosis (59.6% of eyes) and cortical opacity (62.4%) were the most common cataract subtypes, while posterior subcapsular cataract was also present in 20.6% of the eyes. Nuclear sclerosis and cortical opacities showed a marked increase in prevalence with advanced age, whereas the prevalence of posterior subcapsular cataract remained comparable between patients younger than 65 and those 65 or older. This pattern aligns with evidence that posterior subcapsular cataract formation is driven predominantly by prolonged systemic corticosteroid exposure—80.6% of our cohort had received steroids—rather than aging alone [16].

Our multimodal assessments demonstrated that OCT retained robust sensitivity for ellipsoid zone disruption regardless of cataract severity. Even in advanced cataracts, outer retinal abnormalities remained discernible, likely because the near-infrared light source of our OCT penetrated lens opacities more effectively than shorter-wavelength modalities. Automated perimetry also maintained detection sensitivity via pattern deviation analyses. Although total deviation maps were depressed by diffuse lens opacity, characteristic parafoveal scotomas were clearly visible on the pattern deviation maps, reinforcing the utility of HVF in assessing cataractous eyes for HCQ retinopathy screening.

By contrast, FAF detection rates were significantly different between mild (92.1%) and clinically significant (61.5%; *p* < 0.001) cataracts, which cause the lens to absorb and scatter more light, reducing contrast in autofluorescence images [17,18,19]. Hyperautofluorescent changes—key in early retinal toxicity—were particularly attenuated, whereas hypoautofluorescent lesions (seen in more advanced disease) were relatively preserved (Figure 4). In the subset of eyes that had undergone cataract surgery, detection rates for FAF increased, demonstrating the reversibility of the sensitivity and suggesting that timely cataract surgery not only restored visual function but also enabled reliable FAF screening.

The results of this study have several important implications for the clinical management of patients on HCQ therapy. First, our results strongly support the use of multimodal imaging for screening, as the reliance on a single modality—particularly FAF in patients with cataracts—may lead to missed cases of early retinopathy [20,21]. OCT appears to be the most reliable imaging modality in patients with cataracts and should be considered the primary screening tool in this population. Furthermore, the high prevalence of cataracts in our cohort highlights the need for comprehensive ophthalmic evaluation, including a detailed lens assessment, in all patients undergoing screening for HCQ retinopathy. The presence and severity of cataracts should be documented and considered when interpreting screening results, particularly FAF images. In patients with clinically significant cataracts, OCT (for structural changes) and HVF pattern deviation (for functional deficits) should be prioritized. FAF remains valuable for detecting advanced toxicity, although it is less reliable for early screening in cataractous eyes. However, the marked improvement in detection capability following cataract surgery suggests that timely cataract extraction may be beneficial in patients with significant lens opacity who require ongoing HCQ therapy and monitoring. This consideration should be discussed in a shared decision-making process with patients, weighing the risks of surgery against the benefits of potentially improved vision and screening accuracy.

This retrospective study was limited by its single-center design and potential selection bias toward patients with more advanced retinopathy and cataract, given that our institution is a tertiary care facility. Furthermore, because we included only patients with confirmed HCQ retinopathy, our findings may overestimate the impact of cataracts on overall screening. A prospective study enrolling all patients on HCQ—both with and without retinopathy—would yield more comprehensive data on the prevalence and severity of cataracts and their influence on detection rates. Additionally, we graded lens opacities using the LOCS III scale rather than objective quantitative densitometry; future studies employing quantitative measurements of lens optical density—such as Scheimpflug-based densitometry—could enable more precise correlations between opacity levels and imaging performance [22,23]. Moreover, cataract grading was performed by a single experienced examiner using the predefined grading scheme (LOCS III). Although this approach minimized inter-grader variability and ensured consistent application of grading criteria, it may introduce single-examiner bias. Independent review by multiple observers and inter-observer validation (e.g., assessment of inter-observer agreement such as Cohen’s kappa) were not possible in this retrospective study and should be addressed in future investigations. We used a swept-source OCT device (~1050 nm), which penetrates lens opacities better than shorter-wavelength spectral-domain OCT (~840 nm). Other SD-OCT systems may be more prone to signal loss. Prospective studies comparing swept-source vs. spectral-domain OCT in cataractous eyes—and exploring the impact of different cataract subtypes on specific FAF excitation/emission spectra—could further refine screening algorithms [24,25]. Several factors may also influence the imaging quality and interpretation beyond cataract severity. Variations in pupil size, mild vitreous haze, or concurrent macular comorbidities (e.g., age-related macular changes) may further degrade FAF or OCT signals and complicate diagnostic interpretation; these factors were not systematically assessed in this retrospective study. Finally, emerging imaging modalities such as near-infrared autofluorescence warrant further investigation for their potential to overcome cataract-related limitations of conventional autofluorescence imaging.

## 5. Conclusions

In conclusion, cataracts significantly impair the FAF-based detection of HCQ retinopathy, but do not substantially affect OCT or HVF. Clinicians should adapt screening strategies accordingly, emphasizing OCT and HVF in patients with cataracts; carefully examine cataract status for interpreting FAF findings; and consider timely cataract removal for a better quality of vision and retinopathy screening. These considerations and approaches will help ensure the early and accurate detection of HCQ toxicity by minimizing the effect of cataracts on HCQ retinopathy screening, ultimately protecting vision in those being treated with the drug.

## Figures and Tables

**Table 1:** Demographic and clinical characteristics of the included patients with hydroxychloroquine retinopathy (*n* = 72).

Characteristics	Mean (SD) or Number
Sex, female (%)	68 (94.4)
Age, yrs	60.8 ± 12.4 (range: 20–81)
Diagnosis, SLE:RA:others *	31:37:4
Best-corrected visual acuity, logMAR	0.26 ± 0.41
Spherical equivalent, diopter (D)	−1.4 ± 3.0
Daily dose, mg	261.8 ± 78.5
Daily dose/real body weight (RBW), mg/kg	5.0 ± 1.5
Daily dose/RBW > 5 mg/kg (%)	30 (41.7)
Duration of hydroxychloroquine use, months	190.6 ± 84.3 (range: 24–372)
Cumulative dose, g	1487 ± 775
Systemic corticosteroid use (%)	58 (80.6)
Severity of retinopathy, eyes	
Early:moderate:severe (%)	50:32:59 (35.5:22.7:41.8)
Pattern of retinopathy, eyes	
Parafoveal:pericentral:mixed (%)	15:83:43 (10.6:58.9:30.5)

SD = standard deviation; SLE = systemic lupus erythematosus; RA = rheumatoid arthritis; logMAR = logarithm of the minimum angle of resolution. * Others include Sjogren syndrome and antiphospholipid syndrome.

**Table 2:** Details of cataracts in the included eyes.

Characteristics (*n* = 141 Eyes)	Number (%)
Nuclear opalescence (LOCS III)	
None or Gr 1	57 (40.4%)
Grade 2	51 (36.2%)
Grade 3	28 (19.9%)
Grade 4	3 (2.1%)
Grade 5	2 (1.4%)
Grade 6	0 (0%)
Cortical opacity (LOCS III)	
None	53 (37.6%)
Grade 1	31 (22.0%)
Grade 2	43 (30.5%)
Grade 3	10 (7.1%)
Grade 4	4 (2.8%)
Grade 5	0 (0%)
Posterior subcapsular cataract (LOCS III)	
None	112 (79.4%)
Grade 1	2 (1.4%)
Grade 2	7 (5.0%)
Grade 3	16 (11.3%)
Grade 4	4 (2.8%)
Grade 5	0 (0%)
Clinically significant cataract	52 (26.9%)
Received cataract surgery after retinopathy diagnosis	23 (16.3%)

**Table 3:** Univariate and multivariate analyses of clinical characteristics associated with detection by fundus autofluorescence (FAF) and Humphrey visual field (HVF) at maximum cataract severity.

Characteristics	FAF				HVF			
	Univariate		Multivariate		Univariate		Multivariate	
	Odds Ratio (95% CI)	*p*	Odds Ratio (95% CI)	*p*	Odds Ratio (95% CI)	*p*	Odds Ratio (95% CI)	*p*
Age		0.732				0.132		
Sex		0.162				0.797		
Medical diagnosis		0.957			2.56 (0.87–7.53)	0.087		0.154
Severity of retinopathy	3.35 (1.84–6.13)	<0.001	4.85 (1.40–16.9)	0.013	4.00 (1.60–10.00)	0.003	3.37 (1.17–9.71)	0.024
Pattern of retinopathy *, Pericentral	0.060 (0.008–0.46)	0.007		0.785	0.13 (0.016–1.01)	0.051		0.472
Cortical opacity grade	0.45 (0.29–0.68)	<0.001	0.43 (0.22–0.85)	0.015		0.131		
Nuclear opalescence grade	0.69 (0.46–1.05)	0.084	0.21 (0.07–0.66)	0.007		0.314		
Posterior subcapsular opacity grade		0.624				0.182		

* Parafoveal as reference.

## Data Availability

The original contributions presented in this study are included in the article/Supplementary Materials. Further inquiries can be directed to the corresponding author.

## Supplementary Materials

The following supporting information can be downloaded at: https://www.mdpi.com/article/10.3390/diagnostics15212736/s1, Figure S1. A flowchart of the study population and inclusion/exclusion criteria used. Figure S2. Photographic examples of FAF (left), HVF (right upper), and OCT (right lower) in a 54-year-old female patient with hydroxychloroquine retinopathy and cataract (LOCS III: C1P4 [inset]) before and after cataract surgery. Figure S3. OCT image quality index by presence of clinically significant cataract. Figure S4. Prevalence of clinically significant cataract for each type in included eyes (*n* = 141).

## Abbreviations

The following abbreviations are used in this manuscript:

ERG	Electroretinogram
FAF	Fundus autofluorescence
HCQ	Hydroxychloroquine
HVF	Humphrey visual field
OCT	Optical coherence tomography
RA	Rheumatoid arthritis
RPE	Retinal pigment epithelium
SLE	Systemic lupus erythematosus
