# Supplementary material for: Effect of Cataracts on Hydroxychloroquine Retinopathy Screening
Source: Diagnostics (Basel). 2025 Oct 28;15(21):2736. doi: 10.3390/diagnostics15212736 (PMC12609966; doi:10.3390/diagnostics15212736)

Supplemental Figure S1. A flowchart of the study population and inclusion/exclusion criteria used

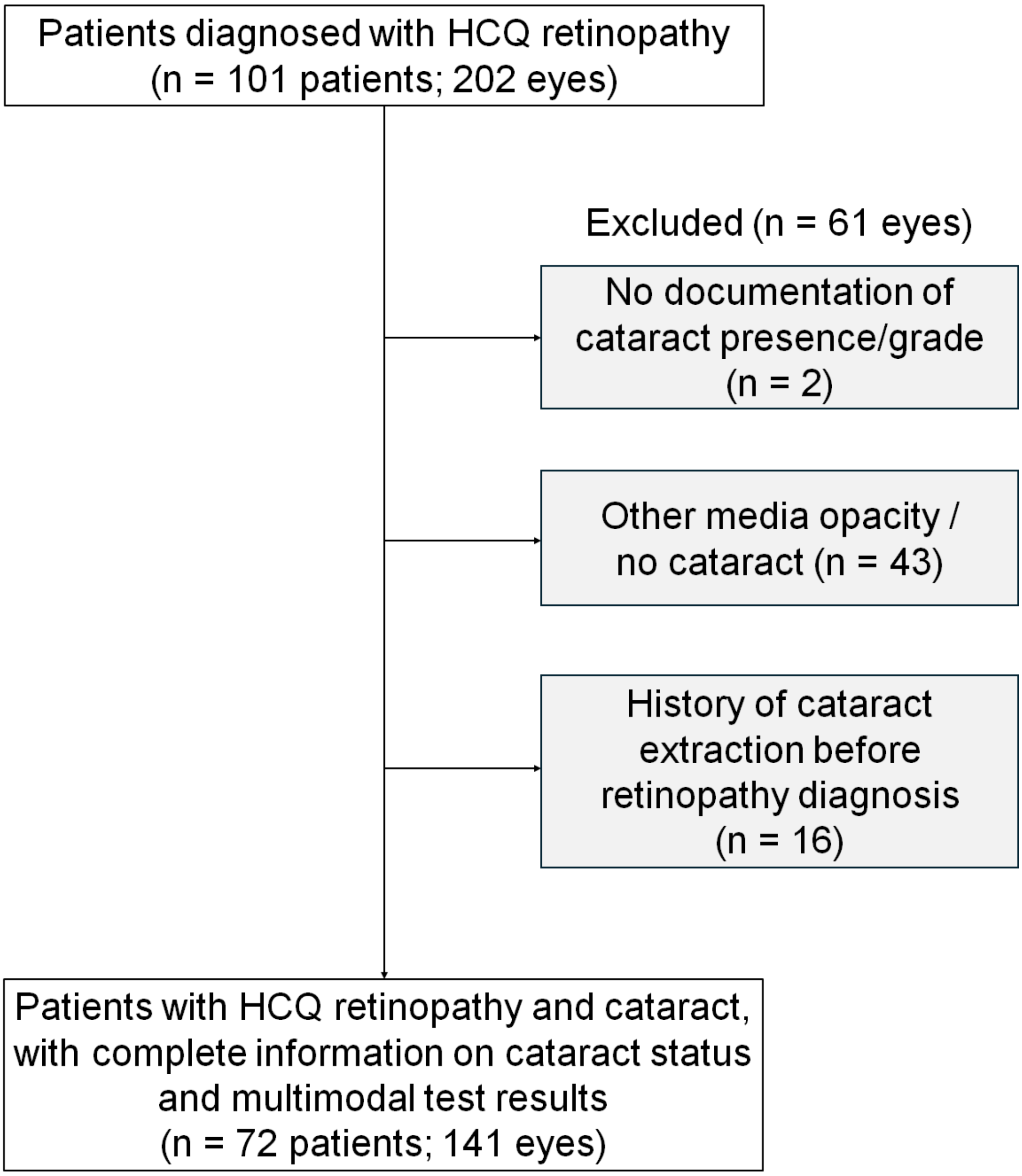

Supplemental Figure S2. Photographic examples of FAF (left), HVF (right upper), and OCT (right lower) in a 54-year-old female patient with hydroxychloroquine retinopathy and cataract (LOCS III: C1P4 [inset]) before and after cataract surgery. OCT is mildly affected but still reveals pericentral ellipsoid zone loss (arrowheads), whereas FAF is severely obscured by a posterior subcapsular cataract, preventing visualization of the posterior pole (arrows). HVF reliably detects abnormalities in the pattern deviation map.

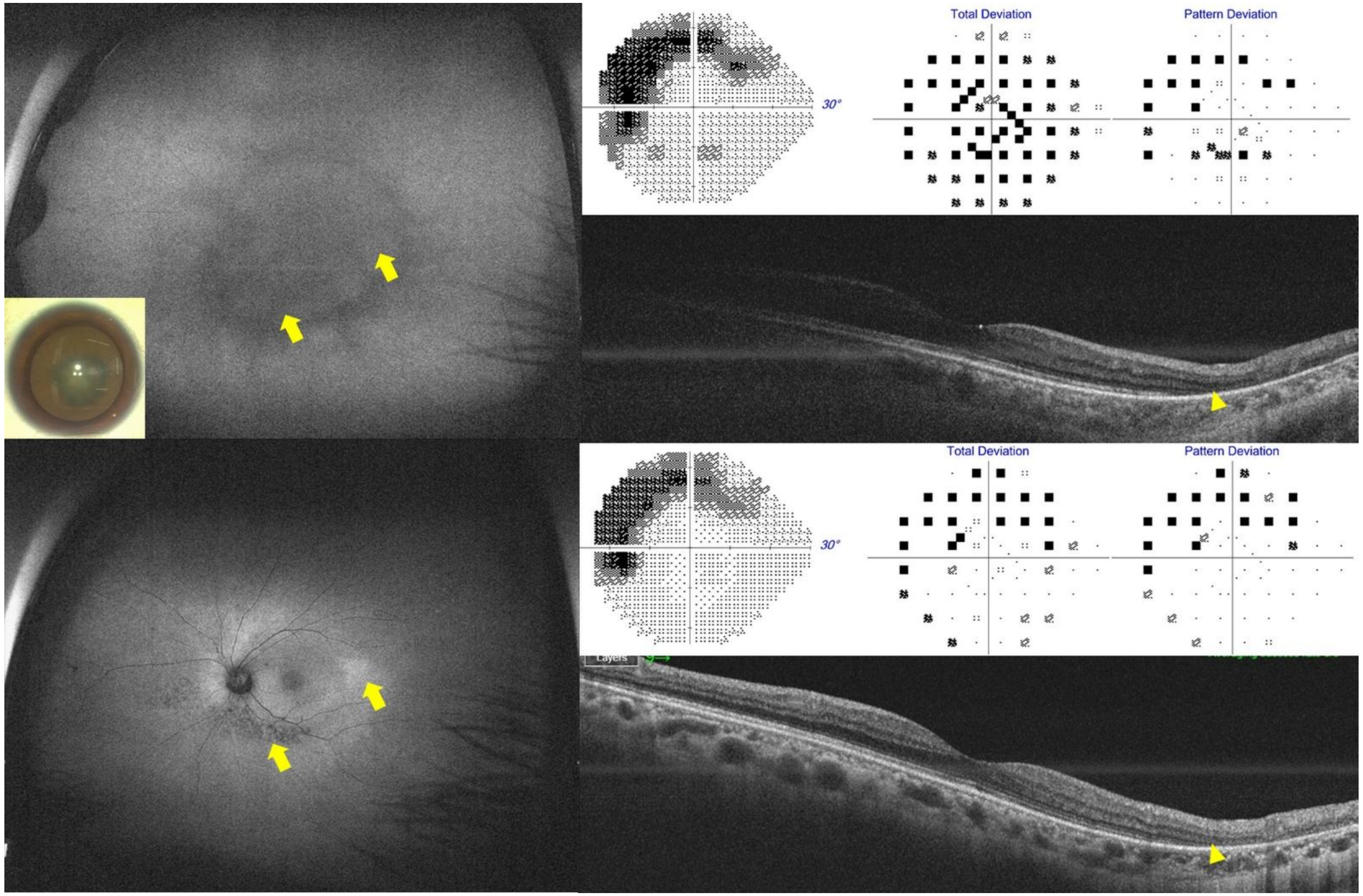

Supplemental Figure S3. OCT image quality index by presence of clinically significant cataract

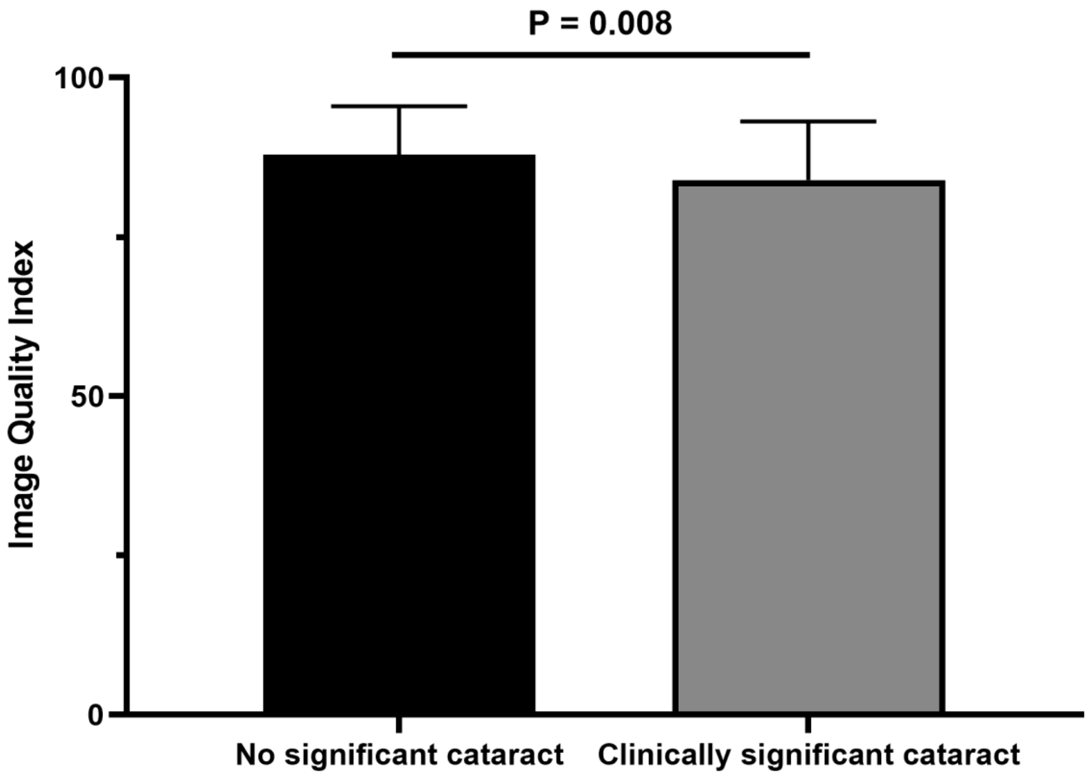

Supplemental Figure S4. Prevalence of clinically significant cataract for each type in included eyes (n = 141).

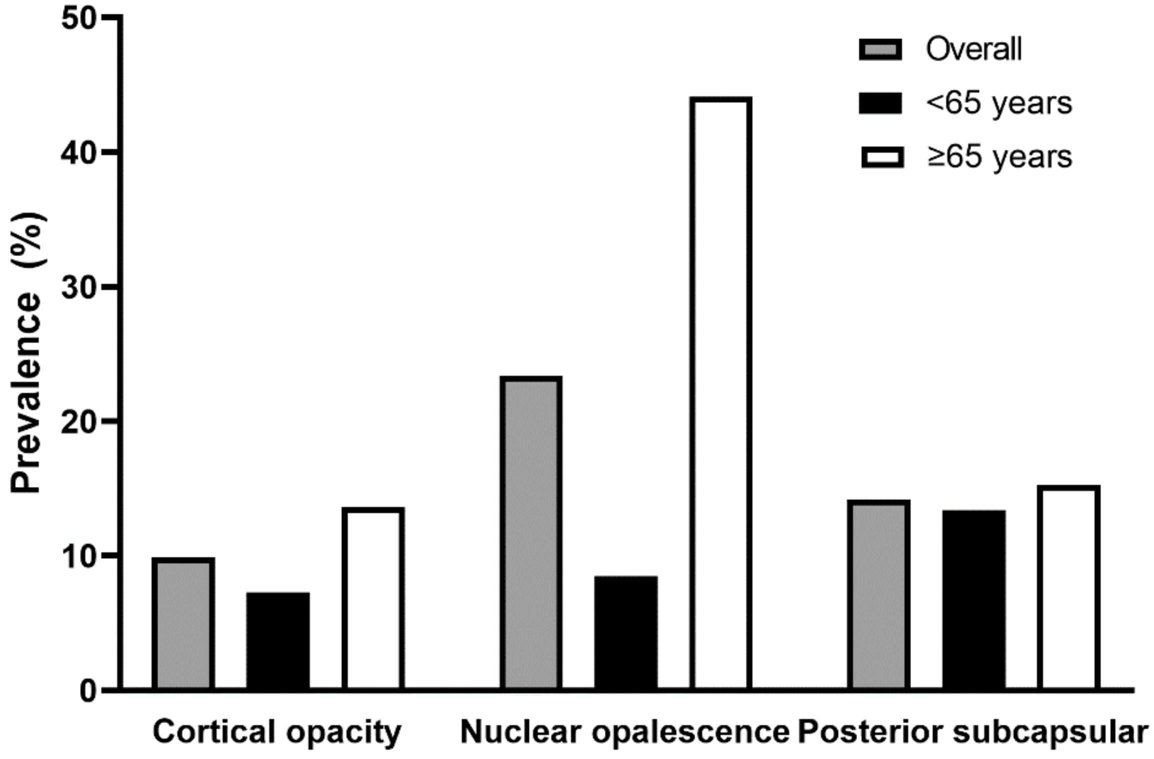

Supplement: Supplementary file 1 [file diagnostics-15-02736-s001.zip › diagnostics-3922629-supplementary.pdf]
